# Predictive Factors of Mortality in Acute Amphetamine Type Stimulants Poisoning; a Review of 226 Cases

**Published:** 2018-01-10

**Authors:** Mitra Rahimi, Somaieh Lookzadeh, Roxana Sadeghi, Kambiz Soltaninejad, Shahin Shadnia, Abdolkarim Pajoumand, Hossein Hassanian-Moghaddam, Nasim Zamani, Masoud Latifi-Pour

**Affiliations:** 1 Toxicology Research Center, Excellence Center of Clinical Toxicology, Department of Clinical Toxicology, Loghman Hakim Hospital, School of Medicine, Shahid Beheshti University of Medical Sciences, Tehran, Iran; 2 Cardiology Department, Loghman Hakim Hospital, School of Medicine, Shahid Beheshti University of Medical Sciences, Tehran, Iran; 3 Department of Forensic Toxicology, Legal Medicine Research Center, Legal Medicine Organization, Tehran, Iran

**Keywords:** Amphetamines, patient outcome, prognostic factors, poisoning, substance abuse

## Abstract

**Introduction:**

Amphetamine type stimulants (ATS) such as amphetamine and methamphetamine (MA) are one of the most important causes of poisoning in the world. In this study we aimed to define the predictive factors of mortality in acute ATS poisoning patients.

**Methods:**

This is a retrospective cross-sectional study on all cases with acute ATS poisoning who were referred to a referral center for poisoning, Tehran, Iran, from April 2011 to March 2014. Using patients^’ ^medical records, demographic data, route of exposure, type and amount of ATS, the cause of poisoning, clinical presentations, and electrocardiogram (ECG) and laboratory findings, as well as patient’s outcomes were collected and analyzed regarding the independent predictive factors of mortality.

**Results:**

226 cases with the mean age of 32.9 ± 10.9 years were studied (77% male). MA was the most abused ATS (97.4%) and the most frequent route of exposure was oral (55.3%). The mortality rate was 5.4%. There was a significant association between agitation (p = 0.002), seizure (p = 0.001), loss of consciousness (p < 0.001), creatine phosphokinase level (p = 0.002), serum pH (p = 0.002), serum HCO_3_ (p = 0.02), and PCO_2_ (p = 0.01) with mortality. However, serum HCO_3 _[OR=1.27 (95% CI: 1.07-1.50); p value=0.005], PCO_2_ [OR=0.89 (95% CI: 0.84-0.96); p value=0.002], and loss of consciousness [OR=0.019 (95% CI: 0.003-0.106); p value=0.000] were the only independent predictive factors of mortality.

**Conclusion:**

PCO_2 _≥ 51 mmHg, serum bicarbonate ≤ 22.6 mEq/L, and loss of consciousness on admission could be considered as prognostic factors of mortality in acute ATS poisoning cases presenting to emergency department.

## Introduction:

Amphetamine type stimulants (ATS) refer to a class of substances whose main derivatives are amphetamine and methamphetamine (MA). Also, a range of other substances such as ephedrine, pseudoephedrine, methylphenidate, methcathinone, and 3,4- methylenedioxymethamphetamine (MDMA) are included in this group (1, 2).

The production and abuse of ATS have increased worldwide. However, the pattern of abuse of each type of ATS is different throughout the world based on social, cultural and geographic parameters (1). For example, amphetamine abuse tends to be more common than MA in Europe, except in Czech Republic, Slovakia, Estonia, and Latvia. Amphetamine is used therapeutically and illicitly in the United Kingdom (3). MA, which is the second most popular illicit drug with an annual global prevalence estimated at 0.4% (3), is more dominant in the east, southeast Asia and Oceania (4). 

ATS abuse is a serious health, social and economic problem all over the world. Previous reports have determined the significant correlation between ATS abuse (mostly MA) via injection and risk of the transmission of blood-borne viruses (3). Dependency and addiction are other mental and clinical health problems among ATS abusers. Dependence and chronic usage are associated with MA psychosis and other related psychosomatic and clinical adverse consequences (3).

Another health problem in ATS abusers is acute poisoning, which is considered as a major problem in emergency settings (5, 6). The common features of ATS poisoning include agitation, dilated pupils, tachycardia, hypertension, and tachypnea. Other clinical findings include tremor, dyspnea, chest pain, hyperpyrexia and cardiac, hepatic and/or renal failure. Coma or seizures occur less frequently (7). 

Although there are reports about acute ATS poisoning (8-11), to the best of our knowledge there are limited data about prognostic factors of mortality in acute ATS poisoning cases (12, 13). Therefore, the aim of the present study was to define the predictive factors of mortality in acute ATS poisoning patients.

## Methods:

***Study design and setting***

This is a retrospective cross-sectional study. All cases of pure acute ATS poisoning, who were referred to the Toxicology Center of Loghman Hakim Hospital, Tehran, Iran, since April 2011 to March 2014, were studied. This educational hospital serves as a referral center for poisoning patients of Iranian capital, Tehran. The study was approved by Ethical Committee of Shahid Beheshti University of Medical Sciences (Grant No.: M-384). The authors adhered to the principles of Helsinki declaration. The patients' data were kept confidential.

***Participants***

All cases of pure acute ATS poisoning who were admitted during the mentioned period were enrolled to the study using census sampling. The patients with co-ingestion or those discharged against medical advice were excluded. 

***Data gathering***

Using a self-made checklist, demographic data (sex, age), route of exposure, type and amount of ATS, the cause of poisoning, history of addiction, clinical presentations, laboratory findings, electrocardiography (ECG) finding, duration of hospitalization, and outcomes (mortality, disposition, and complications during admission) were collected by a trained physician for all participants according to the patients^’^ medical records. We used ICD10 classification for extracting patients^’^ medical records from the hospital’s archive. Diagnosis of acute ATS poisoning was done based on the history given by the patients or their relatives, physical examination and laboratory confirmation.

***Statistical analysis***

 We used the social package for statistical analysis (SPSS) software version 16. The data were expressed as mean ± SD for continuous or discrete variables and as frequency and percentage for categorical variables. Chi-square test was used for statistical analysis of qualitative variables. The normal distribution of quantitative variables was tested by Kolmogorov – Smirnov test. The statistical comparison was done with Mann–Whitney U -test for nonparametric variables and independent student t-test for parametric variables. Logistic regression was used for evaluating the predictive factors of mortality. The best cut off points was determined by calculating the area under the receiver operating characteristics (ROC) curve. P values of 0.05 or less were considered to be statistically significant and data were presented with 95% confidence interval (CI).

## Results:

***Baseline characteristics***

**Diagram 1:**
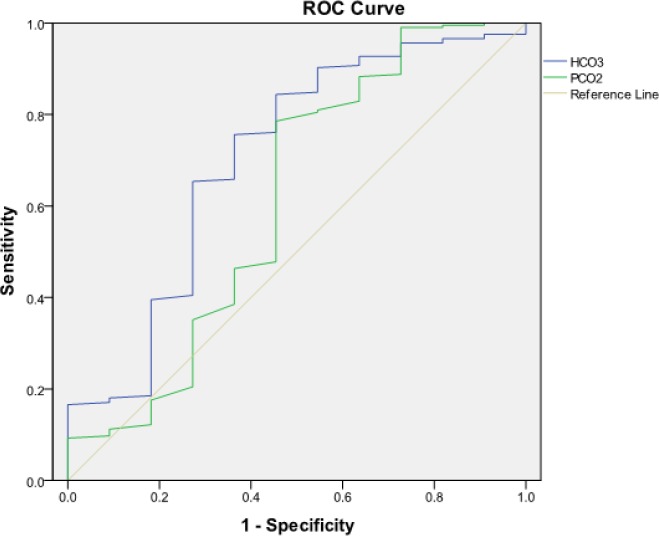
Flow diagram of patient selection. ATS: Amphetamine Type Stimulants

1722 ATS intoxicated patients^’ ^files were evaluated, out of which, 226 (13%) cases with acute ATS poisoning were included (Diagram 1). The mean age of the patients was 32.9 ± 10.9 (14 - 77) years (77% male). Baseline characteristics, clinical presentations, and laboratory results, as well as ECG findings are summarized in table 1 and 2. 

The most common type of ATS used was MA (97.4%) and the most frequent route of exposure was oral (55.3%). Abuse was the most common cause of poisoning (66.8%) and the mean ATS dose was 1.64 ± 1.59 grams. The mean time from exposure to admission was 5.9 ± 9.6 hours. History of addiction was positive in 123 (54%) cases with 5.3 ± 3.8 years mean duration of addiction.

**Table 1:** Comparing the baseline characteristics of acute amphetamine type stimulants (ATS) intoxicated patients who survived and those who died

**Parameter**	**Total (n=226)**	**Survived (n=214)**	**Died (n=12)**	**P value**
**Sex**				
Male	174 (77)	165 (77.1)	9 (75)	0.6
Female	52 (23)	49 (22.9)	3 (25)	
**Type of ATS**				
Methamphetamine	220 (97.4)	208 (97.2)	12 (100)	0.9
MDMA	1 (0.4)	1 (0.5)	0	
Methylphenidate	5 (2.2)	5 (2.3)	0	
**Route of exposure**				
Oral	125 (55.3)	116 (54.2)	9 (75)	0.7
Inhalation	93 (41.2)	90 (42)	3 (25)	
Injection	4 (1.8)	4 (1.9)	0	
Oral and Inhalation	3 (1.3)	3 (1.4)	0	
Oral and Injection	1 (0.4)	1 (0.5)	0	
**Cause of poisoning**				
Abuse	151 (66.8)	146 (68.2)	5 (41.7)	0.07
Suicide	54 (23.9)	47 (22)	7 (58.3)	
Accidental	2 (0.9)	2 (0.9)	0	
Body packer	15 (6.6)	15 (7)	0	
Body stuffer	4 (1.8)	4 (1.9)	0	
**ATS dose (gram)**	1.64±1.59 (0.5-13)	1.7±1.6 (0.5-13)	1.1±0.3 (1-1.5)	0.8
**Exposure to admission (hour)**	5.9±9.6 (0.5-72)	6±9.8 (0.5-72)	4.4±3.1 (1-10)	0.9
**Duration of addiction (year)**	5.3±3.8 (1-20)	5.3±3.9 (1-20)	5.5±3.1 (3-10)	0.9
**Age (year)**	32.9±10.9 (14-77)	32.6±10.7 (14-77)	38.1±13.6 (19-55)	0.2

Data were presented as mean ± standard deviation (minimum-maximum) or frequency (%). MDMA: 3,4-Methylendeoxymethamphetamine.

**Table 2:** Comparing the vital signs, clinical presentations, laboratory results, and electrocardiogram (ECG) findings among acute amphetamine type stimulants (ATS) intoxicated patients who survived and those who died

**Parameter **	**Total (n=226)**	**Survived (n=214)**	**Died (n=12)**	**P**
**Vital signs**				
SBP (mmHg)	125.5±23.6 (80-230)	126±22.7 (80-230)	115.8±36.3 (80-180)	0.1
DBP (mmHg)	78.5±13.9 (40-150)	78.9±13.7 (50-150)	70±16.5 (40-100)	0.054
Pulse rate (/minute)	99.2±19 (52-168)	99.2±18.5 (52-168)	99.8±27.1 (66-160)	0.6
**Clinical presentations**				
Agitation	172 (76.11)	168 (78.50)	4 (33.33)	0.002
Confusion	66 (29.20)	65 (30.37)	1 (8.33)	0.09
Judgment disorder	48 (21.24)	48 (22.43)	0	0.053
Seizure	16 (7.08)	11 (5.14)	5 (41.67)	0.001
LOC	16 (7.08)	9 (4.21)	7 (58.33)	0.000
Hallucination	14 (6.19)	13 (6.0)	1(8.3)	0.6
Diaphoresis	12 (5.31)	12 (5.31)	0	0.5
Flushing	7 (3.1)	7 (3.1)	0	0.7
Abdominal pain	5 (2.21)	5 (2.21)	0	0.8
Blurred vision	2 (0.88)	2 (0.88)	0	0.9
**Laboratory findings**				
Sodium (mEq/L)	140.1±5.0 (124-188)	140.1±5.0 (124-188)	140.1±5.1 (130-148)	1
Potassium (mEq/L)	4.1±0.5 (3-7.7)	4.1±0.5 (3.1-7.7)	4.2±0.9 (3-5.9)	0.5
CPK (U/L)	1067.9±2981.9 (28-30000)	813.2±1952.4 (28-17253)	7309.1±10263.3 (103-30000)	0.002
LDH (U/L)	909.1±841.3 (42-6033)	885.7±854.6 (42-6033)	1225±635.2 (563-2043)	0.1
Serum pH	7.36±0.09 (6.90-7.90)	7.36±0.09 (6.90-7.90)	7.27±0.15 (6.90-7.40)	0.002
PCO2 (mmHg)	43.9±9.8 (13-78)	43.6±9.2 (13-74)	51.2±16.5 (32-78)	0.01
Serum HCO3 (mEq/L)	24.3±4.9 (8.7-56)	24.5±4.9 (8.7-56)	21.2±4.5 (15.8-28.4)	0.02
**ECG findings**				
Normal sinus	104 (46)	97 (45.3)	7 (58.3)	0.3
Sinus tachycardia	103 (45.6)	100 (46.7)	3 (25.0)	
Sinus bradycardia	8 (3.5)	7 (3.2)	1 (8.3)	
T inversion	10 (4.4)	9 (4.2)	1 (8.3)	
QRS widening	7 (3.0)	6 (2.8)	1 (8.3)	
ST change	4 (1.7)	3 (1.4)	1 (8.3)	
Ventricular Dysrhythmia	3 (1.3)	2 (0.9)	1 (8.3)	
**Hospitalization (hour)**				
≤24	139 (61.6)	132 (61.7)	7 (58.3)	0.07
>24	87 (38.4)	82 (38.3)	5 (41.7)	

Data were presented as mean ± standard deviation (minimum-maximum) or frequency (%). SBP: Systolic blood pressure, LOC: Loss of consciousness, DBP: Diastolic blood pressure, CPK: Creatine Phosphokinase, LDH: Lactate dehydrogenase; ECG: Electrocardiogram.

**Table 3:** Screening performance Characteristics of PCO_2_ ≥ 51 mmHg and serum HCO_3_ ≤ 22.6 mEq/L in predicting the risk of mortality in acute amphetamine Type Stimulants (ATS) intoxicated patients

**Character**	**PCO2 (95% CI)**	**HCO3 (95% CI)**
**Sensitivity**	50.00 (22.28 – 77.71)	66.66 (35.43 – 88.72)
**Specificity**	78.97 (72.77 – 84.10)	72.89 (66.33 – 78.62)
**Positive Predictive Value**	11.76 (04.87 – 24.55)	12.12 (05.74 – 23.03)
**Negative Predictive Value**	96.57 (92.34 – 98.59)	97.50 (93.31 – 99.19)
**Positive Likelihood Ratio**	0.13 (0.06 – 0.28)	0.13 (0.07 – 0.26)
**Negative Likelihood Ratio**	0.03 (0.01 - 0.07)	0.02 (0.01 - 0.06)

**Figure 1:**
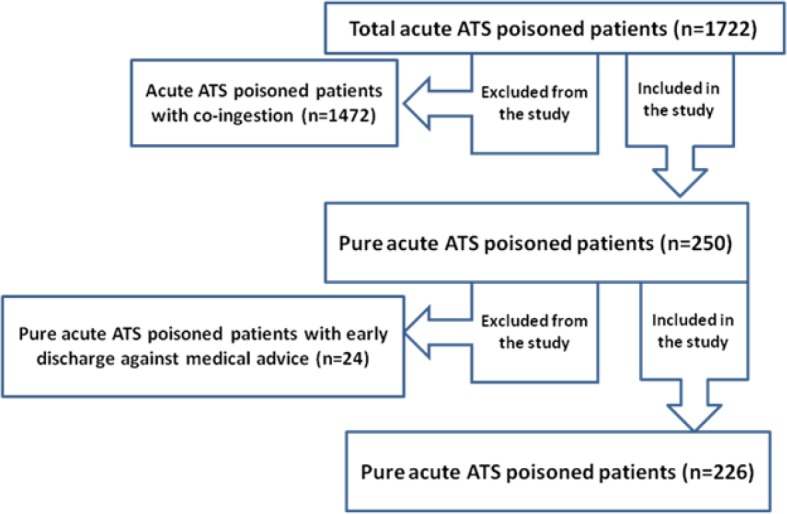
Receiver operating characteristic (ROC) curve for PCO_2 _and serum HCO_3_ in predicting the risk of mortality in acute amphetamine Type Stimulants (ATS) intoxicated patients.

***Outcomes***

19 (8.4%) cases were admitted to Medical Toxicology Intensive Care Unit (ICU) and others (207, 91.6% of cases) were admitted to general ward. In most of the cases (61.6%), the duration of hospitalization was ≤ 24 hours. Intubation was indicated in 24 (10.6%) cases. The mortality rate was 5.4% (12/226). 2 cases of acute respiratory distress syndrome (0.9%), 2 ventilator associated pneumonia (0.9%), 2 rhabdomyolysis (0.9%), and 1 case of deep vein thrombosis (0.4%) were the complications detected in 7 (3.1%) patients (All of them were male). 

***Predictive factors of mortality***

Table 1 and 2 compare the baseline characteristics, clinical presentations, laboratory results, and ECG findings of acute ATS poisoning among survived and non-survived cases. 

Based on univariate analysis, there were significant associations between agitation (p = 0.002), seizure (p = 0.001), loss of consciousness on admission (p < 0.001), creatine phosphokinase level (p = 0.002), serum pH (p = 0.002), serum HCO_3_ (p = 0.02), and PCO_2_ (p = 0.01) with mortality. 

However, the results of multivariate regression analysis showed serum HCO_3 _[OR=1.27 (95% CI: 1.07-1.50); p value=0.005], PCO_2_ [OR=0.89 (95% CI: 0.84-0.96); p value=0.002], and loss of consciousness on admission [OR=0.019 (95% CI: 0.003-0.106); p value=0.000] as the independent predictive factors of mortality in acute ATS poisoning. 

Based on the area under the ROC curve (AUC) the best cut off points of PCO_2_ and serum HCO_3_ for prediction of mortality were ≥ 51 mmHg [AUC = 0.61 (95% CI: 0.401-0.822)] and ≤ 22.6 mEq/L [AUC = 0.704 (95% CI: 0.525-0.882)], respectively (figure 1). 

Screening performance characteristics of PCO2 ≥ 51 mmHg and HCO_3_ ≤ 22.6 mEq/L in prediction of acute ATS intoxicated mortality are summarized in table 3.

## Discussion:

ATS are potent psychostimulants that are abused all over the world (4). ATS poisoning has recently emerged as a crucial health problem in clinical and forensic settings (8, 14, 15). Therefore, the emergency department staff should be aware of the clinical presentations, paraclinical findings and prognostic factors of acute ATS poisoning. 

In this study, the most common cause of poisoning was abuse and majority of cases, had oral exposure. In the previous study done in the same hospital, although the main cause of poisoning was abuse, the common route of exposure was inhalation (12). 

In our study, most of the patients were young men, which is in concordance with the results of previous studies (6, 12, 13). Most of our patients had a positive history of addiction. This result is in line with previous studies (6, 12). 

MA was the most frequent type of ATS used by our patients. The result is the same as previous studies in Iran (12, 16), however, the studies in European countries showed amphetamine and MDMA as the most frequent type of ATS among intoxicated cases (10, 17). This difference could be due to demographic variables such as marital status and level of education (18). Previous studies introduced curiosity in trying different things and looking for pleasure as the most important reasons for MA abuse in Iran (18, 19). Other factors are lower effectiveness of previous drugs, popularity and low price of new drugs, and emulation of others (18, 20, 21). Khodabandeh et al. reported MA abuse among methadone maintenance participants. The most common reasons were the good sensation, getting high, to enhance their sexual performance, and in some instances as self-medication for depression (22, 23). 

Agitation, confusion and judgment disorder were the most common clinical manifestations. which are the same as the result of a previous study in Australia (6). In another study in Iran, loss of consciousness was the most common clinical finding, which could be due to co-ingestion with opioids and other drugs (12).

Most of the patients had abnormal ECG, and sinus tachycardia was the major finding, which has been reported previously (12, 24). 

The mortality rate was 5.4%, which was lower than previous studies (12, 13). This may be related to the delayed admission of the patients to the hospital in the previous studies (12).

In our study, lower serum HCO_3_, higher PCO_2_ and low level of consciousness on admission were considered as predicting factors of mortality in ATS poisoning cases. In the previous study performed in this center, age, history of suicide, route of poisoning and pulmonary manifestations on admission were considered as predictive factors of patient's outcome (12), which is not supported by the results of our study.

According to our results, PCO_2_≥ 51 mmHg and serum HCO_3 _≤ 22.6 mEq/L can predict the poisoned patients’ mortality rate with specificity (78.97% and 72.89%, respectively) and sensitivity (50.00% and 66.66%, respectively). However, based on the AUC measures, serum HCO_3_ can better discriminate between those who die and those who survive. We did not find a study that had evaluated the relationship between these laboratory findings and prediction of mortality. 

## Limitations:

We evaluated the patients’ records retrospectively, which could be considered as a limitation of our study.

## Conclusion:

This study showed that high PCO_2_, low serum bicarbonate and loss of consciousness on admission, could be associated with higher rate of mortality of acute ATS intoxicated cases. 
